# Novel transcriptome resources for three scleractinian coral species from the Indo-Pacific

**DOI:** 10.1093/gigascience/gix074

**Published:** 2017-08-23

**Authors:** Carly D. Kenkel, Line K Bay

**Affiliations:** 1 Australian Institute of Marine Science, PMB No 3, Townsville MC, Queensland 4810, Australia;; 2 Department of Biological Sciences, University of Southern California, 3616 Trousdale Parkway, Los Angeles, CA 90089, USA

**Keywords:** *Galaxea astreata*, *Galaxea acrhelia*, *Goniopora columna*, thermal stress, functional genomics

## Abstract

Transcriptomic resources for coral species can provide insight into coral evolutionary history and stress-response physiology. *Goniopora columna, Galaxea astreata*, and *Galaxea acrhelia* are scleractinian corals of the Indo-Pacific, representing a diversity of morphologies and life-history traits. *G. columna* and *G. astreata* are common and cosmopolitan, while *G. acrhelia* is largely restricted to the coral triangle and Great Barrier Reef. Reference transcriptomes for these species were assembled from replicate colony fragments exposed to elevated (31°C) and ambient (27°C) temperatures. Trinity was used to create *de novo* assemblies for each species from 92–102 million raw Illumina Hiseq 2 × 150 bp reads. Host-specific assemblies contained 65 460–72 405 contigs, representing 26 693–37 894 isogroups (∼genes) with an average N50 of 2254. Gene name and/or gene ontology annotations were possible for 58% of isogroups on average. Transcriptomes contained 93.1–94.3% of EuKaryotic Orthologous Groups comprising the core eukaryotic gene set, and 89.98–91.92% of the single-copy metazoan core gene set orthologs were complete, indicating fairly comprehensive assemblies. This work expands the complement of transcriptomic resources available for scleractinian coral species, including the first reference for a representative of *Goniopora* spp. as well as species with novel morphology.

## Data Description

### Background

A growing body of genomic information for reef-building corals has resolved phylogenetic relationships and helped reveal how this unique taxonomic group calcifies and responds to thermal stress [1–4]. Such information is critical for understanding the adaptive capacity of these ecologically important organisms, particularly in an era of global climate change [5]. Transcriptomic and/or genomic resources are currently available for 23 scleractinian species representing 14 genera and 11 families [1, 4, 6–16]. We assembled the transcriptomes of 3 scleractinian coral species: the congeners *Galaxea astreata, G. acrhelia*, and *Goniopora columna*. This is the first sequence resource for *Goniopora* spp. and extends the phenotypic diversity represented by coral transcriptomic resources to include submassive (*G. astreata*) and columnar (*G. columna*) morphologies [17], which should facilitate additional insight into the evolutionary history of this taxonomic order.

### Samples and sequencing

Samples of *Galaxea astreata* and *Galaxea acrhelia* were collected from Davies Reef (18°49.816’S, 147°37.888’E) on 8–11 April 2015, and samples of *Goniopora columna* were collected from Pandora Reef (18°48.778’S, 146°25.593’E) on 20–22 April 2015 under Great Barrier Reef Marine Park Authority permit G12/35 236.1 and G14/37 318.1.

To generate more comprehensive reference transcriptomes, 4–5 replicate cores of a single colony were subjected to a 2-week temperature stress experiment as described in Kenkel and Bay (2017) [18], and paired samples from control (27°C) and heat (31°C) treatments were snap-frozen in liquid nitrogen on day 2, day 4, and day 17 (Table 1; note for *G. acrhelia*, heat-treated fragments were only included for day 4 and day 17). Samples were crushed in liquid nitrogen, and total RNA was extracted using an Aurum Total RNA mini kit (Bio-Rad, Irvine, CA, USA). RNA quality and quantity were assessed using the NanoDrop ND-200 UV-Vis Spectrophotometer (Thermo Scientific, Waltham, MA, USA) and gel electrophoresis.

**Table 1:** Assembly statistics for *de novo* transcriptomes by coral species

	*Galaxea astreata*	*Galaxea acrhelia*	*Goniopora columna*
N heat	3	2	3
N ctrl	2	2	2
N raw reads (×10^6^)	92.8	96.0	102.8
N qual filtered: PE, SE (×10^6^)	35.0, 5.8	33.3, 6.0	26.9, 4.7
N contigs holobiont	173 883	164 996	185 625
N contigs host only	65 460	67 127	72 405
Mean GC content host only	42.3%	42.1%	42.2%
N isogroups	29 145	26 693	37 894
Mean contig length (bp)	1754	1894	1492
N50 (bp)	2300	2480	1984
Contiguity at 0.75	0.40	0.41	0.37
% annotated	62.4	60.7	50.1
% core KOGs	94.3	94.0	93.1
BUSCOs			
N complete (%)	880 (89.98%)	899 (91.92%)	881 (90.08%)
N partial (%)	36 (3.68%)	30 (3.07%)	31 (3.17%)
N missing (%)	62 (6.34%)	49 (5.01%)	66 (6.75%)

For transcriptome sequencing, RNA samples from replicate fragments were pooled in equal proportions, and ∼1 μg was shipped on dry ice to the Oklahoma Medical Research Foundation NGS Core, where Illumina TruSeq Stranded libraries were prepared and sequenced on 1 lane of the Illumina Hiseq 3000/4000 to generate 2 × 150 PE reads.

### Transcriptome assembly and annotation

Sequencing yielded 92–102 million raw PE reads (Table 1). The *fastx_toolkit* [19] was used to discard reads <50 bp or having a homopolymer run of “A” ≥9 bases, retain reads with a PHRED quality of at least 20 over 80% of the read, and to trim TruSeq sequencing adaptors. Polymerase chain reaction duplicates were then removed using a custom perl script [20]. Remaining high-quality filtered reads (26–35 million paired reads, 4–6 million unpaired reads) (Table 1) were assembled using Trinity v. 2.0.6 (Trinity, RRID:SCR_013048) [21] using the default parameters and an *in silico* read normalization step at the Texas Advanced Computing Center at the University of Texas at Austin.

Since corals are “holobionts” comprised of host, *Symbiodinium*, and other microbial components, resulting assemblies were filtered to identify the host component following the protocol described in Kitchen et al. (2015) [4], with one modification. Briefly, small clusters (= contigs, <400bp) were removed, and a hierarchical series of blast searches against potential contaminants was conducted. First, assemblies were compared to the most complete Cnidarian rRNA database (SILVA: ABAV01023297, ABAV01023333) [22] using BLASTn [23], and good matches (bit-score >45) were removed. Next, transcriptomes were compared to a Cnidarian mitochondrial genome using BLASTn (*Acropora tenuis*, NCBI: NC_0 03522.1) [24], again discarding contigs with match bit-scores >45. The taxonomic origin of remaining contigs was identified using a series of BLASTx searches against the most complete coral and *Symbiodinium* gene models (coral: *Acropora digitifera*, adi_v1.01_prot, [14]; *Symbiodinium*: *S. kawagutii*, Symbiodinium_kawagutii.0819.final.gene.pep, [25]) and NCBI’s nonredundant (nr) protein database (downloaded 25 July 2016) [23]. For a contig to remain in the host-specific assembly, it had to both match (E value ≤ 10^−5^) a gene in the coral proteome more closely than the *Symbiodinium* proteome and match a metazoan sequence or have no match in the nr database. In addition, contigs with no match to either proteome were also retained if they exhibited a best match to a Cnidarian in the nr database search, a slightly less stringent criterion than that used by Kitchen et al. (2015) [4]. Annotation of host transcriptomes was performed following the protocols and scripts described in [26]. Host contigs were assigned putative gene names and gene ontologies using a BLASTx search (E value ≤ 10^−4^) against the UniProt Knowledgebase Swiss-Prot database [27]. EuKaryotic Orthologous Groups (KOG) annotations were assigned using a BLAST search against the core eukaryotic gene set from the CEGMA pipeline (CEGMA, RRID:SCR_015055) [28] and the WebMGA server (WebMGA, RRID:SCR_011951; [29]) [30] and Kyoto Encyclopedia of Genes and Genomes (KEGG) IDs using the KAAS server [31, 32]. The stats.sh command of the BBMap package [33] was used to calculate GC content of host transcriptomes. Transcriptome completeness was evaluated through comparison to the Benchmarking Universal Single-Copy Ortholog v. 2 (BUSCO, RRID:SCR_015008) [34] set for metazoans using the gVolante server [35, 36].

### Evaluation of assemblies

The initial holobiont assemblies contained 164 996–185 625 contigs over 400 bp in length (N_50_ = 1543–1848). Of these, 34–94 were discarded as matching non-mRNAs (9–10 rRNA, 25–74 mitochondrial). Following screening for biological contamination, 64 249–68 968 contigs had a best match to the *Acropora digitifera* proteome, and of these, 59 875–65 367 matched either a metazoan or had no match in NCBI’s nr database. An additional 5585–7038 contigs matched neither proteome but exhibited a best hit to a Cnidarian in the nr database and were also retained. These host-specific assemblies represented 26 693–37 894 isogroups (∼genes) with an average length of 1492–1894 bp and an N50 of 1984–2480 (Table 1). Mean GC content of host-specific assemblies was 42% (Table 1), which is consistent with other anthozoan transcriptomes where *Symbiodinium* reads have been effectively filtered [16]. Protein coverage exceeded 0.75 for 37–41% of contigs (Table 1). Gene name and/or gene ontology annotations were possible for 16 196–19 306 (50.1–62.4%) of these isogroups based on sequence homology comparisons to the Swiss-Prot database (Table 1) [27]. KEGG pathway annotation [32] resulted in 4488–4728 unique matches for 7105–8712 isogroups. Comparison of these assemblies to the core eukaryotic 248-gene set [28] revealed that 93.1–94.3% of KOGs were represented, and annotation of isogroups resulted in 23–24 unique KOG matches for 8700–10 025 isogroups (Table 1). Of the 978 core BUSCO gene sets for metazoans [34], 89.98–91.92% were found to be complete, while an additional 3.07–3.68% were partially assembled, indicating that assemblies are fairly comprehensive (Table 1).

### Re-use potential

These coral host-specific assemblies are sufficient for use as transcriptome references for Tag-based RNAseq (TagSeq) [37], a cost-effective method that was recently shown to be more accurate at quantifying gene expression levels than traditional RNAseq [38]. The fasta files and associated annotation files have been formatted for direct use in the TagSeq read mapping [39] and GO-MWU analysis pipelines [40].

## Data accessibility

Raw reads are archived at NCBI’s SRA under project numbers PRJNA350363: *Goniopora columna*; PRJNA352640: *Galaxea archelia*; PRJNA352641: *Galaxea astreata*. Transcriptomes, annotation files, and other supporting data are available via the *Gigascience* repository, *Giga*DB [41]. The assembled transcriptomes and associated annotation files can also be obtained from http://dornsife.usc.edu/labs/carlslab/data/ or from the Australian Institute of Marine Science Data Centre at http://data.aims.gov.au/metadataviewer/faces/view.xhtml?uuid=3c2d31c9-b921–491c-ae27–0d169fa98c84.

## Abbreviations

KEGG: Kyoto Encyclopedia of Genes and Genomes; KOG: EuKaryotic Orthologous Groups; TagSeq: Tag-based RNAseq.

## Funding

Funding for this study was provided by an National Science Foundation International Postdoctoral Research Fellowship, DBI-1 401 165, to C.D.K. and funding from the Australian Institute of Marine Science to C.D.K. and L.K.B.

## Competing interests

The authors have no competing interests to declare.

## Author contributions

C.D.K. conceived and designed the experiments; C.D.K. and L.K.B. performed the experiments; C.D.K. performed bioinformatics analyses and wrote the first draft. L.K.B. contributed to revisions and read and approved the final manuscript.

## Supplementary Material

GIGA-D-17-00060_Original-Submission.pdf

GIGA-D-17-00060_Revision-1.pdf

GIGA-D-17-00060_Revision-2.pdf

Response-to-Reviewer-Comments_Original-Submission.pdf

Response-to-Reviewer-Comments_Revision-1.pdf

Reviewer-1-Report-(Original-Submission).pdf

Reviewer-2-Report-(Original-Submission).pdf

Reviewer-2-Report-(Revision-1).pdf

## References

[1] Bhattacharya D, Agrawal S, Aranda M Comparative genomics explains the evolutionary success of reef-forming corals. eLife 2016;5:e13288 doi: http://dx.doi.org/10.7554/eLife.13288. 27218454 10.7554/eLife.13288 PMC4878875

[2] Dixon GB, Davies SW, Aglyamova GV Genomic determinants of coral heat tolerance across latitudes. Science 2015;348(6242):1460–2. 26113720 10.1126/science.1261224

[3] Bay A, Palumbi R Multilocus adaptation associated with heat resistance in reef-building corals. Curr Biol 2014;24(24):2952–6. 25454780 10.1016/j.cub.2014.10.044

[4] Kitchen SA, Crowder CM, Poole AZ De novo assembly and characterization of four Anthozoan (Phylum Cnidaria) transcriptomes. G3 Genes Genomes Genet 2015;5:2441–52. 10.1534/g3.115.020164 PMC4632063 26384772

[5] Hughes TP, Baird AH, Bellwood DR Climate change, human impacts, and the resilience of coral reefs. Science 2003;301(5635):929–33. 12920289 10.1126/science.1085046

[6] Davies SW, Marchetti A, Ries JB Thermal and pCO2 stress elicit divergent transcriptomic responses in a resilient coral. Front Mar Sci 2016;3. doi: 10.3389/fmars.2016.00112.

[7] Moya A, Huisman L, Ball EE Whole transcriptome analysis of the coral *Acropora millepora* reveals complex responses to CO 2-driven acidification during the initiation of calcification. Mol Ecol 2012;21(10):2440–54. 22490231 10.1111/j.1365-294X.2012.05554.x

[8] Kenkel CD, Meyer E, Matz MV Gene expression under chronic heat stress in populations of the mustard hill coral (*Porites astreoides*) from different thermal environments. Mol Ecol 2013;22(16):4322–34. 23899402 10.1111/mec.12390

[9] Shinzato C, Inoue M, Kusakabe M A snapshot of a coral holobiont: a transcriptome assembly of the scleractinian coral, porites, captures a wide variety of genes from both the host and symbiotic zooxanthellae. PLoS One 2014;9(1):e85182 doi:10.1371/journal.pone.0085182. 24454815 10.1371/journal.pone.0085182 PMC3893191

[10] Anderson DA, Walz ME, Weil E RNA-Seq of the Caribbean reef-building coral *Orbicella faveolata* (Scleractinia-Merulinidae) under bleaching and disease stress expands models of coral innate immunity. Peer J 2016;4:e1616 doi:https://doi.org/10.7717/peerj.1616. 26925311 10.7717/peerj.1616 PMC4768675

[11] Traylor-Knowles N, Granger BR, Lubinski TJ Production of a reference transcriptome and transcriptomic database (PocilloporaBase) for the cauliflower coral, *Pocillopora damicornis*. BMC Genomics 2011;12(1):585. 22126435 10.1186/1471-2164-12-585 PMC3339375

[12] Barshis DJ, Ladner JT, Oliver TA Genomic basis for coral resilience to climate change. Proc Natl Acad Sci U S A 2013;110(4):1387–92. 23297204 10.1073/pnas.1210224110 PMC3557039

[13] Polato NR, Vera JC, Baums IB Gene discovery in the threatened elkhorn coral: 454 sequencing of the *Acropora palmata* transcriptome. PLoS One 2011;6(12):e28634 doi:10.1371/journal.pone.0028634. 22216101 10.1371/journal.pone.0028634 PMC3247206

[14] Shinzato C, Shoguchi E, Kawashima T Using the *Acropora digitifera* genome to understand coral responses to environmental change. Nature 2011;476(7360):320–3. 21785439 10.1038/nature10249

[15] Libro S, Kaluziak ST, Vollmer SV RNA-seq profiles of immune related genes in the staghorn coral *Acropora cervicornis* infected with white band disease. PLoS One 2013;8(11):e81821 doi:10.1371/journal.pone.0081821. 24278460 10.1371/journal.pone.0081821 PMC3836749

[16] Lin Z, Chen M, Dong X Transcriptome profiling of *Galaxea fascicularis* and its endosymbiont Symbiodinium reveals chronic eutrophication tolerance pathways and metabolic mutualism between partners. Sci Rep 2017;7:42100 doi:10.1038/srep42100. 28181581 10.1038/srep42100 PMC5299600

[17] Madin JS, Anderson KD, Andreasen MH The Coral Trait Database, a curated database of trait information for coral species from the global oceans. Sci Data 2016;3:160017 doi:10.1038/sdata.2016.17. 27023900 10.1038/sdata.2016.17 PMC4810887

[18] Kenkel CD, Bay LK The role of vertical symbiont transmission in altering cooperation and fitness of coral-Symbiodinium symbioses. BioRxiv 2017 doi: https://doi.org/10.1101/067322.

[19] http://hannonlab.cshl.edu/fastx_toolkit.

[20] https://github.com/ckenkel/annotatingTranscriptomes.

[21] Grabherr MG, Haas BJ, Yassour M Full-length transcriptome assembly from RNA-seq data without a reference genome. Nat Biotechnol 2011;29(7):644–52. 21572440 10.1038/nbt.1883 PMC3571712

[22] Quast C, Pruesse E, Yilmaz P The SILVA ribosomal RNA gene database project: improved data processing and web-based tools. Nucleic Acids Res 2013;41(D1):D590–6. 23193283 10.1093/nar/gks1219 PMC3531112

[23] Altschul SF, Gish W, Miller W Basic local alignment search tool. J Mol Biol 1990;215(3):403–10. 2231712 10.1016/S0022-2836(05)80360-2

[24] Van Oppen MJH, Catmull J, McDonald BJ The mitochondrial genome of *Acropora tenuis* (Cnidaria; Scleractinia) contains a large group I intron and a candidate control region. J Mol Evol 2002;55(1):1–13. 12165838 10.1007/s00239-001-0075-0

[25] Lin S, Cheng S, Song B The *Symbiodinium kawagutii* genome illuminates dinoflagellate gene expression and coral symbiosis. Science 2015;350(6261):691–4. 26542574 10.1126/science.aad0408

[26] https://github.com/ckenkel/annotatingTranscriptomes.

[27] The UniProt Consortium UniProt: a hub for protein information. Nucleic Acids Res 2015;43:D204–12. 25348405 10.1093/nar/gku989 PMC4384041

[28] Parra G, Bradnam K, Korf I CEGMA: a pipeline to accurately annotate core genes in eukaryotic genomes. Bioinformatics 2007;23:1061–7. 17332020 10.1093/bioinformatics/btm071

[29] http://weizhong-lab.ucsd.edu/metagenomic-analysis/server/kog/.

[30] Wu S, Zhu Z, Fu L WebMGA: a customizable web server for fast metagenomic sequence analysis. BMC Genomics 2011;12:444 doi:10.1186/1471-2164-12-444. 21899761 10.1186/1471-2164-12-444 PMC3180703

[31] http://www.genome.jp/kegg/kaas/.

[32] Moriya Y, Itoh M, Okuda S KAAS: an automatic genome annotation and pathway reconstruction server. Nucleic Acids Res 2007;35:W182–5. 17526522 10.1093/nar/gkm321 PMC1933193

[33] Bushnell B BBMap Short Read Aligner. Berkeley, CA: University of California, Berkeley, 2016.

[34] Simão FA, Waterhouse RM, Ioannidis P BUSCO: assessing genome assembly and annotation completeness with single-copy orthologs. Bioinformatics 2015; doi:10.1093/bioinformatics/btv351. 10.1093/bioinformatics/btv351 26059717

[35] https://gvolante.riken.jp/analysis.html.

[36] Nishimura O, Hara Y, Kuraku S gVolante for standardizing completeness assessment of genome and transcriptome assemblies. Bioinformatics 2017;3210 doi: 10.1093/bioinformatics/btx445. 10.1093/bioinformatics/btx445 PMC5870689 29036533

[37] Meyer E, Aglyamova GV, Matz MV Profiling gene expression responses of coral larvae (*Acropora millepora*) to elevated temperature and settlement inducers using a novel RNA-Seq procedure. Mol Ecol 2011;20:3599–616. 21801258 10.1111/j.1365-294X.2011.05205.x

[38] Lohman BK, Weber JN, Bolnick DI Evaluation of TagSeq, a reliable low-cost alternative for RNAseq. Mol Ecol Resour 2016; doi:10.1111/1755-0998.12529. 10.1111/1755-0998.12529 27037501

[39] https://github.com/z0on/tag-based_RNAseq.

[40] https://github.com/z0on/GO_MWU.

[41] Kenkel CD, Bay LK Supporting data for “Novel transcriptome resources for three scleractinian coral species from the Indo-Pacific.” GigaScience Database 2017 http://dx.doi.org/10.5524/100328. 10.1093/gigascience/gix074 PMC5603760 28938722

