# Supplementary material for: Novel transcriptome resources for three scleractinian coral species from the Indo-Pacific
Source: Gigascience. 2017 Aug 23;6(9):1–4. doi: 10.1093/gigascience/gix074 (PMC5603760; doi:10.1093/gigascience/gix074)
Supplement: GIGA-D-17-00060_Original-Submission.pdf [file gix074_GIGA-D-17-00060_Original-Submission.pdf]

Manuscript

Click here to download Manuscript GigaScienceDataNote_v2.docx

Novel transcriptome resources for three scleractinian coral species from the Indo-Pacific

Carly D Kenkel[a,1] and Line K Bay[a,2]

[a]Australian Institute of Marine Science, PMB No 3, Townsville MC, Queensland 4810, Australia

[1]Corresponding author, email: carly.kenkel@gmail.com or c.kenkel@aims.gov.au; phone: +61 07 4753 4268; fax: +61 07 4772 5852

[2]Email: L.Bay@aims.gov.au

ABSTRACT

Background

Transcriptomic resources for coral species can provide insight into coral evolutionary history and stress-response physiology. *Goniopora columna*, *Galaxea astreata* and *Galaxea acrhelia* are scleractinian corals of the Indo-Pacific, representing a diversity of morphologies and life-history traits. *G. columna* and *G. astreata* are common and cosmopolitan, while *G. acrhelia* is largely restricted to the coral triangle and Great Barrier Reef. Reference transcriptomes for these species were assembled from replicate colony fragments exposed to elevated (31°C) and ambient (27°C) temperature.

Findings

Trinity was used to create *de novo* assemblies for each species from 92-102 million raw Illumina Hiseq 2 x 150 bp reads. Host-specific assemblies contained 65,460-72,405 contigs, representing 20,093-31,693 isogroups (~genes) with an average N50 of 2,254. Gene name and/or gene ontology annotations were possible for 61% of isogroups on average and transcriptomes contained 93.1-94.3% of KOGs comprising the core eukaryotic gene set, indicating fairly comprehensive assemblies.

Conclusions

This work expands the complement of transcriptomic resources available for scleractinian coral species, including the first the first reference for a representative of *Goniopora* spp. as well as species with novel morphology.

KEYWORDS: *Galaxea astreata*, *Galaxea acrhelia*, *Goniopora columna*, thermal stress, functional genomics

DATA DESCRIPTION

Background

A growing body of genomic information for reef-building corals has resolved phylogenetic relationships and helped reveal how this unique taxonomic group calcifies and responds to thermal stress [1-4]. Such information is critical for understanding the adaptive capacity of this ecologically important group of organisms, particularly in an era of global climate change [5]. Transcriptomic and/or genomic resources are currently available for 23 scleractinian species representing 14 genera and 11 families [1, 4, 6-16]. We assembled the transcriptomes of three scleractinian coral species: the congeners *Galaxea astreata* and *G. acrhelia* and *Goniopora columna*. This is the first sequence resource for *Goniopora* spp. and extends the phenotypic diversity represented by coral transcriptomic resources to include submassive (*G. astreata*) and columnar (*G. columna*) morphologies [17], which should facilitate additional insight into the evolutionary history of this taxonomic order.

Samples and sequencing

Samples of *Galaxea astreata* and *Galaxea acrhelia* were collected from Davies Reef (18°49.816'S, 147°37.888'E) on the 8-11 April 2015 and samples of *Goniopora columna* from Pandora Reef (18°48.778'S, 146°25.593'E) on the 20-22 April 2015 under Great Barrier Reef Marine Park Authority permit G12/35236.1 and G14/37318.1.

To generate reference transcriptomes, 4-5 replicate cores of a single colony were subject to a two-week temperature stress experiment as described in [18] and paired samples from control (27°C) and heat (31°C) treatments were snap frozen in liquid nitrogen on day 2, day 4 and day 17 (Table 1, note for *G. acrhelia*, heat-treated fragments only included for day 4 and day 17). Samples were crushed in liquid nitrogen and total RNA was extracted using an Aurum Total RNA mini kit (Biorad, CA). RNA quality and quantity were assessed using the

NanoDrop ND-200 UV-Vis Spectrophotometer (Thermo Scientific, MA) and gel electrophoresis.

For transcriptome sequencing, RNA samples from replicate fragments were pooled in equal proportions and ~1 µg was shipped on dry ice to the Oklahoma Medical Research Foundation NGS Core where Illumina TruSeq Stranded libraries were prepared and sequenced on one lane of the Illumina Hiseq 3000/4000 to generate 2 x 150 PE reads.

Transcriptome assembly and annotation

Sequencing yielded 92-102 million raw PE reads (Table 1). The *fastx_toolkit* (http://hannonlab.cshl.edu/fastx_toolkit) was used to discard reads < 50 bp or having a homopolymer run of 'A' ≥ 9 bases, retain reads with a PHRED quality of at least 20 over 80% of the read and to trim TruSeq sequencing adaptors. PCR duplicates were then removed using a custom perl script (https://github.com/z0on/annotatingTranscriptomes). Remaining high quality filtered reads (26-35 million paired reads, 4-6 million unpaired reads, Table 1) were assembled using Trinity v 2.0.6 [19] using the default parameters and an *in silico* read normalization step at the Texas Advanced Computing Center (TACC) at the University of Texas at Austin.

Since corals are 'holobionts' comprised of host, *Symbiodinium* and other microbial components, resulting assemblies were filtered to identify the host component following the protocol described in Kitchen *et al.* [4], with one modification. Briefly, small clusters (=contigs, <400bp) were removed and a hierarchical series of blast searches against potential contaminants was conducted. First, assemblies were compared to the most complete Cnidarian rRNA database (SILVA: ABAV01023297, ABAV01023333, [20]) using BLASTn [21] and good matches (bit-score > 45) were removed. Next, transcriptomes were compared to a Cnidarian mitochondrial genome using BLASTn (Acropora tenuis, NCBI: NC_003522.1 [22]), again discarding contigs with match bit-scores > 45. The taxonomic origin of

remaining contigs was identified using a series of BLASTx searches against the most complete coral and *Symbiodinium* gene models (coral: *Acropora digitifera*, adi_v1.01_prot, [14]; *Symbiodinium*: *S. kawagutii*, Symbiodinium_kawagutii.0819.final.gene.pep, [23]) and NCBI's nonredundant (nr) protein database (downloaded 25 July 2016, Altschul 1990). For a contig to remain in the host-specific assembly, it had to both match (E value <= 10^{-5}) a gene in the coral proteome more closely than the *Symbiodinium* proteome and match a metazoan sequence or have no match in the nr database. In addition, contigs with no match to either proteome were also retained if they exhibited a best match to a Cnidarian in the nr database search, a slightly less stringent criteria than that used by Kitchen et al. [4].

Annotation of host transcriptomes was performed following the protocols and scripts described at https://github.com/z0on/annotatingTranscriptomes. Host contigs were assigned putative gene names and gene ontologies using a BLASTx search (E value <= 10^{-4}) against the UniProt Knowledgebase Swiss-Prot database [24]. KOG (EuKaryotic Orthologous Groups) annotations were assigned using a BLAST search against the core eukaryotic gene set from the CEGMA pipeline [25] and the WebMGA server (http://weizhong-lab.ucsd.edu/metagenomic-analysis/server/kog/, [26] and KEGG (Kyoto Encyclopedia of Genes and Genomes) id's using the KAAS server (http://www.genome.jp/kegg/kaas/ [27]). The stats.sh command of the BBMap package [cite] was used to calculate GC content of host transcriptomes.

Evaluation of assemblies

The initial holobiont assemblies contained 164,996-185,625 contigs over 400 bp in length (N_{50} = 1543-1848). Of these, 34-94 were discarded as matching non-mRNAs (9-10 rRNA, 25-74 mitochondrial). Following screening for biological contamination, 64,249-68,968 contigs had a best match to the *Acropora digitifera* proteome, and of these, 59,875-65,367 matched either a metazoan or had no match in NCBI's nr database. An additional

5,585-7,038 contigs matched neither proteome, but exhibited a best hit to a Cnidarian in the nr databse and were also retained. These host-specific assemblies represented 20,093-31,693 isogroups (~genes) with an average length of 1,492-1,894 bp and an N50 of 1,984-2,480 (Table 1). Mean GC content of host-specific assemblies was 42% (Table 1), which is consistent with other anthozoan transcriptomes where *Symbiodinium* reads have been effectively filtered [16]. Protein coverage exceeded 0.75 for 37-41% of contigs (Table 1). Gene name and/or GO annotations were possible for 12,789-14,563 (53.5-66%) of these isogroups based on sequence homology comparisons to the Swiss-Prot database [24] (Table 1). Comparison of this assembly to the core eukaryotic 248-gene set [25] revealed 93.1-94.3% of KOGs were represented and annotation of isogroups resulted in 23-24 unique KOG matches for 7234-9123 isogroups (Table 1). KEGG pathway annotation [27] resulted in 3,665-4,234 unique matches for 5,260-7,120 isogroups and visual comparison to the *Nematostella vectensis* reference pathways indicate a majority of core metabolic functions are represented (e.g. Fig. S1-3).

DATA ACCESSIBILITY

Raw reads are archived at NCBI's SRA under project numbers PRJNA350363: *Goniopora columna*, PRJNA352640: *Galaxea archelia*, PRJNA352641: *Galaxea astreata*. The assembled transcriptomes and associated annotation files can be obtained from http://dornsife.usc.edu/labs/carlslab/data/.

ACKNOWLEDGEMENTS

A Bouriat was instrumental in performing temperature stress experiments. S Noonan, V Mocellin, A Severati and M Nayfa helped with coral collection and P Muir provided advice on taxonomic identification. Bioinformatic analyses were carried out using computational resources of the Texas Advanced Computer Center (TACC).

FUNDING

Funding for this study was provided by an NSF International Postdoctoral Research Fellowship, DBI-1401165 to CDK and funding from the Australian Institute of Marine Science to CDK and LKB.

AUTHOR CONTRIBUTIONS

CDK conceived and designed the experiments; CDK and LKB performed the experiments; CDK performed bioinformatics analyses and wrote the first draft. LKB contributed to revisions, read, and approved the final manuscript.

DECLARATIONS

The authors have no competing interests to declare.

Table 1. Assembly statistics for *de novo* transcriptomes by coral species.

	Galaxea astreata	*Galaxea acrhelia*	*Goniopora columna*
**N heat**	3	2	3
**N ctrl**	2	2	2
**N raw reads ($x10^6$)**	92.8	96.0	102.8
**N qual filtered: PE, SE ($x10^6$)**	35.0, 5.8	33.3, 6.0	26.9, 4.7
**N contigs holobiont**	173,883	164,996	185,625
**N contigs host only**	65,460	67,127	72,405
**Mean GC content host only**	42.3%	42.1%	42.2%
**N isogroups**	23,548	20,093	31,693
**Mean contig length (bp)**	1,754	1,894	1,492
**N50 (bp)**	2,300	2,480	1,984
**Contiguity at 0.75**	0.40	0.41	0.37
**% Annotated**	66.0	63.6	53.5
**% core KOGs**	94.3	94.0	93.1

Supplementary Material: Novel transcriptome resources for three scleractinian coral species from the Indo-Pacific

Carly D Kenkel[a,1] and Line K Bay[a,2]

[a]Australian Institute of Marine Science, PMB No 3, Townsville MC, Queensland 4810, Australia

[1]Corresponding author, email: carly.kenkel@gmail.com or c.kenkel@aims.gov.au; phone: +61 07 4753 4268; fax: +61 07 4772 5852

[2]Email: L.Bay@aims.gov.au

KEYWORDS: *Galaxea astreata*, *Galaxea acrhelia*, *Goniopora columna*, thermal stress, functional genomics

Figure S1. Mapping of KEGG-annotated isogroups from the *Galaxea astreata* transcriptome to the glycolysis pathway in the fully sequenced genome of the sea anemone *Nematostella vectensis*. Shading indicates presence of the gene in the *G. astreata* assembly.

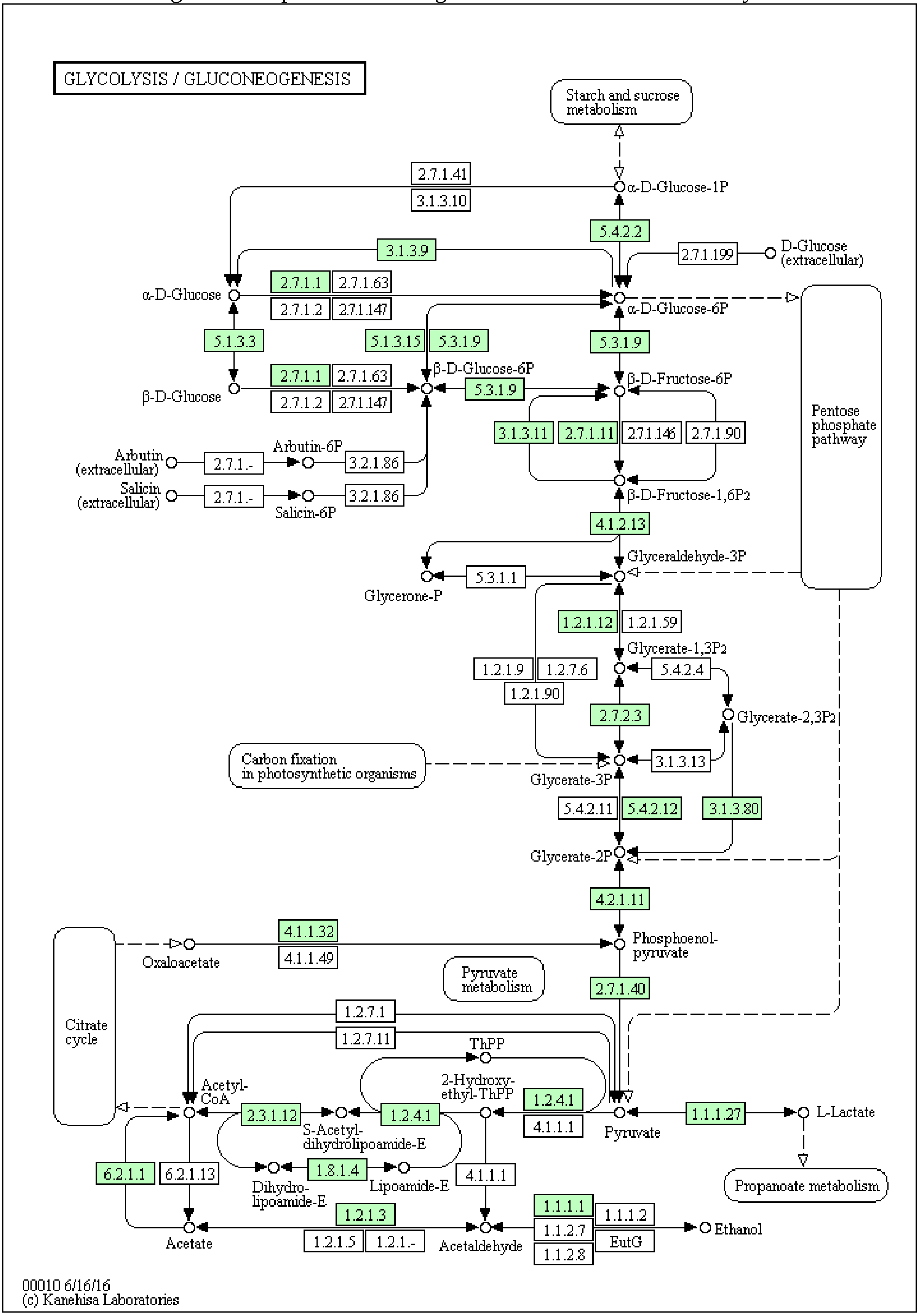

Figure S2. Mapping of KEGG-annotated isogroups from the *Galaxea acrhelia* transcriptome to the glycolysis pathway in the fully sequenced genome of the sea anemone *Nematostella vectensis*. Shading indicates presence of the gene in the *G. acrhelia* assembly.

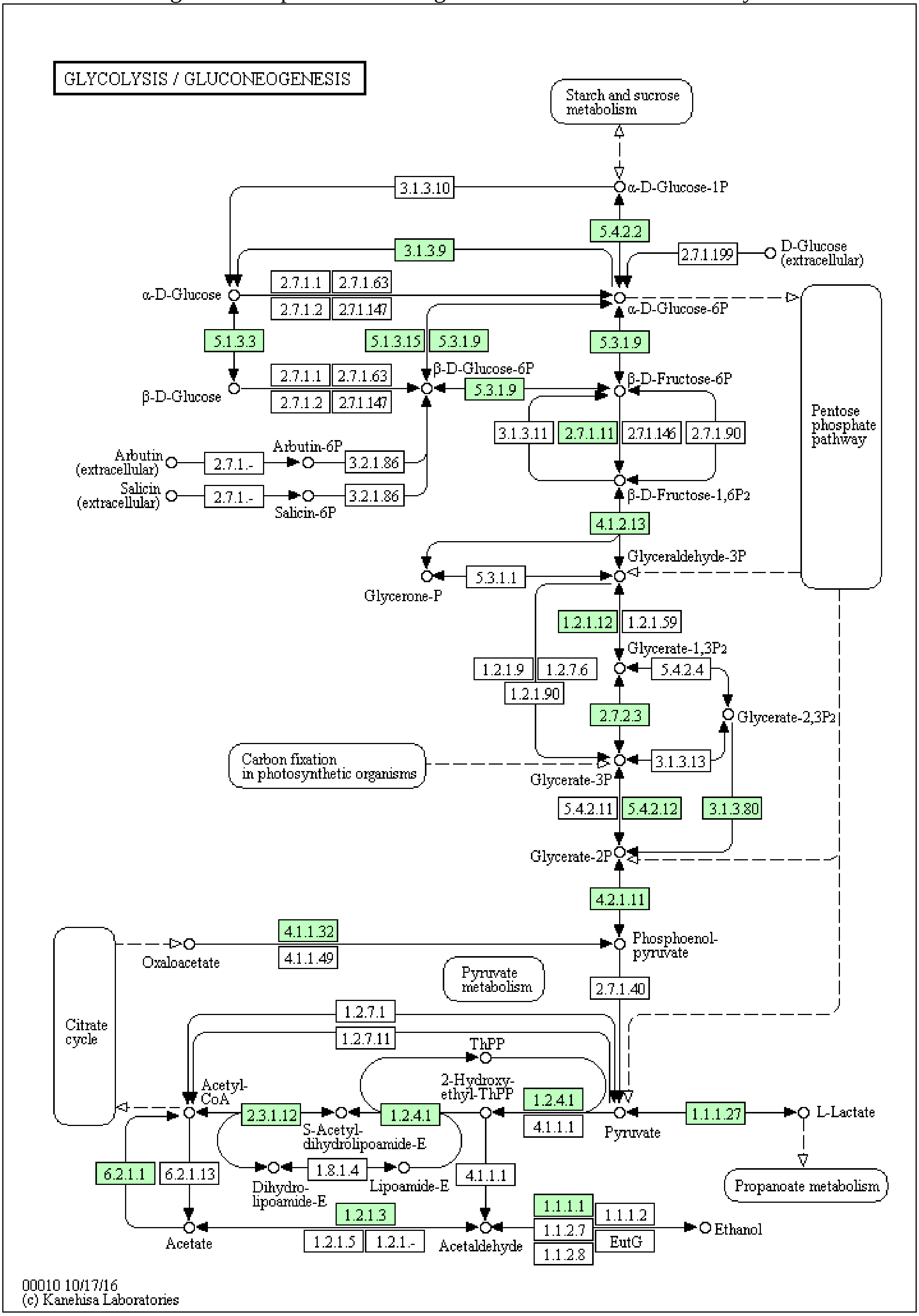

Figure S3. Mapping of KEGG-annotated isogroups from the *Goniopora columna* transcriptome to the glycolysis pathway in the fully sequenced genome of the sea anemone *Nematostella vectensis*. Shading indicates presence of the gene in the *G. columna* assembly.

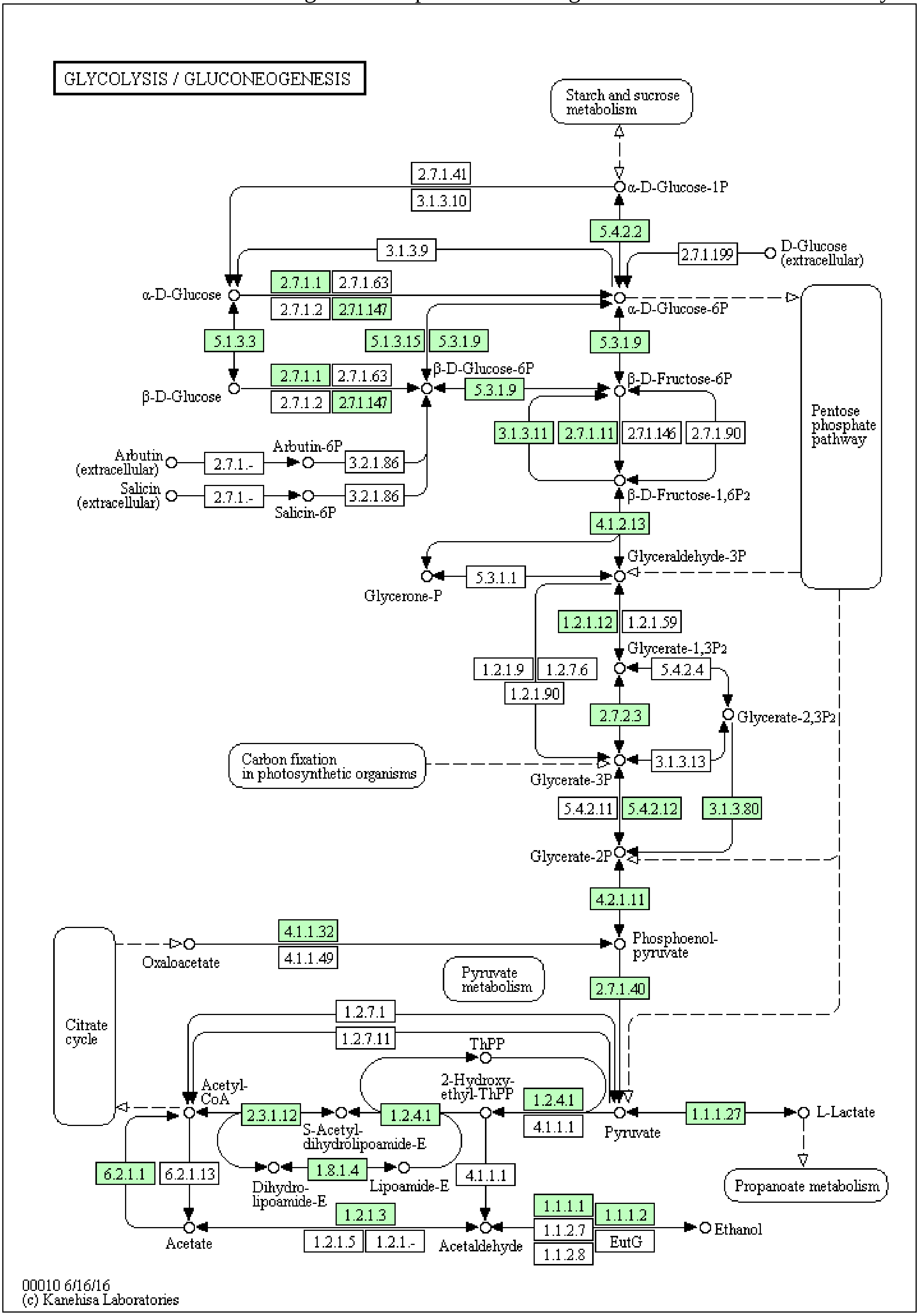

REFERENCES

1. Bhattacharya D, Agrawal S, Aranda M, Baumgarten S, Belcaid M, Drake JL, et al. Comparative genomics explains the evolutionary success of reef-forming corals. eLife. 2016;5:e13288. doi:http://dx.doi.org/10.7554/eLife.13288.
2. Dixon GB, Davies SW, Aglyamova GV, Meyer E, Bay LK and Matz MV. Genomic determinants of coral heat tolerance across latitudes. Science. 2015;348:1460-2.
3. Bay RA and Palumbi SR. Multilocus adaptation associated with heat resistance in reef-building corals. Current Biology. 2014;24:2952-6.
4. Kitchen SA, Crowder CM, Poole AZ, Weis VM and Meyer E. De novo assembly and characterization of four Anthozoan (Phylum Cnidaria) transcriptomes. G3 Genes Genomes Genetics. 2015;5:2441-52. doi:10.153/g3.115.020164.
5. Hughes TP, Baird AH, Bellwood DR, Card M, Connolly SR, Folke C, et al. Climate change, human impacts, and the resilience of coral reefs. Science. 2003;301 5635:929-33.
6. Davies SW, Marchetti A, Ries JB and Castillo KD. Thermal and pCO2 stress elicit divergent transcriptomic responses in a resilient coral. Frontiers in Marine Science. 2016;3 doi:10.3389/fmars.2016.00112.
7. Moya A, Huisman L, Ball EE, Hayward DC, Grasso LC, Chua CM, et al. Whole transcriptome analysis of the coral Acropora millepora reveals compex responses to CO2-driven acidification during the initiation of calcification. Molecular Ecology. 2012;21:2440-54. doi:doi: 10.1111/j.1365-294X.2012.05554.x.
8. Kenkel C, Meyer E and Matz M. Gene expression under chronic heat stress in populations of the mustard hill coral (Porites astreoides) from different thermal environments. Molecular Ecology. 2013;22 16:4322-34. doi:10.1111/mec.12390.
9. Shinzato C, Inoue M and Kusakabe M. A snapshot of a coral "holobiont": A transcriptome assembly of the scleractinian coral, Porites, captures a wide variety of genes from both the host and symbiotic zooxanthellae. PLoS ONE. 2014;9 1:e85182. doi:doi:10.1371/journal.pone.0085182.
10. Anderson DA, Walz ME, Weil E, Tonellato P and Smith MC. RNA-Seq of the Caribbean reef-building coral Orbicella faveolata (Scleractinia-Merulinidae) under bleaching and disease stress expands models of coral innate immunity. PeerJ. 2016;4:e1616. doi:https://doi.org/10.7717/peerj.1616.
11. Traylor-Knowles N, Granger BR, Lubinski TJ, Parikh JR, Garamszegi S, Xia Y, et al. Production of a reference transcriptome and transcriptomic database (PocilloporaBase) for the cauliflower coral, Pocillopora damicornis. BMC Genomics. 2011;12:585.
12. Barshis D, Ladner JT, Oliver TA, Seneca FO, Traylor-Knowles N and Palumbi SR. Genomic basis for coral resilience to climate change. Proceedings of the National Academy of Sciences of the United States of America. 2013;110:1387-92.
13. Polato NR, Vera JC and Baums IB. Gene discovery in the threatened elkhorn coral: 454 sequencing of the Acropora palmata transcriptome. PLoS ONE. 2011;6 12: e28634. doi:10.1371/journal.pone.0028634.
14. Shinzato C, Shoguchi E, Kawashima T, Hamada M, Hisata K, Tanaka M, et al. Using the Acropora digitifera genome to understand coral responses to environmental change. Nature. 2011;476:320-3.

15. Libro S, Kaluziak ST and Vollmer SV. RNA-seq profiles of immune related genes in the staghorn coral Acropora cervicornis infected with white band disease. PLoS ONE. 2013;8 11:e81821. doi:10.1371/journal.pone.0081821.
16. Lin Z, Chen M, Dong X, Zheng X, Huang H, Xu X, et al. Transcriptome profiling of Galaxea fascicularis and its endosymbiont Symbiodinium reveals chronic eutrophication tolerance pathways and metabolic mutualism between partners. Scientific Reports. 2017;7:42100. doi:10.1038/srep42100.
17. Madin JS, Anderson KD, Andreasen MH, Bridge TCL, Cairns SD, Connolly SR, et al. The Coral Trait Database, a curated database of trait information for coral species from the global oceans. Scientific Data. 2016;3:160017. doi:10.1038/sdata.2016.17.
18. Kenkel CD and Bay LK. The role of vertical symbiont transmission in altering cooperation and fitness of coral-Symbiodinium symbioses. BioRxiv doi: https://doiorg/101101/067322. 2017.
19. Grabherr M, Haas B, Yassour M, Levin J, Thompson D, Amit I, et al. Full-length transcriptome assembly from RNA-seq data without a reference genome. Nature Biotechnology. 2011;29 7:644-52.
20. Quast C, Pruesse E, Yilmaz P, Gerken J, Schweer T, Yarza P, et al. The SILVA ribosomal RNA gene database project: improved data processing and web-based tools. Nucleic Acids Research. 2013;41:D590-D6. doi: 10.1093/nar/gks1219.
21. Altschul S, Gish W, Miller W, Myers E and Lipman D. Basic local alignment search tool. Journal of Molecular Biology. 1990;215:403-10.
22. van Oppen MJH, Catmull J, McDonald B, Hislop N, Hagerman P and Miller DJ. The mitochondrial genome of Acropora tenuis (Cnidaria; Scleractinia) contains a large group I intron and a candidate control region. Journal of Molecular Evolution. 2002;55 1:1-13.
23. Lin S, Cheng S, Song B, Zhong X, Lin X, Li W, et al. The Symbiodinium kawagutii genome illuminates dinoflagellate gene expression and coral symbiosis. Science. 2015;350 6261:691-4. doi:10.1126/science.aad0408.
24. Consortium TU. UniProt: a hub for protein information. Nucleic Acids Research. 2015;43:D204-12.
25. Parra G, Bradnam K and Korf I. CEGMA: a pipeline to accurately annotate core genes in eukaryotic genomes. Bioinformatics. 2007;23:1061-7. doi:10.1093/bioinformatics/btm071.
26. Wu S, Zhu Z, Fu L, Niu B and Li W. WebMGA: a customizable web server for fast metagenomic sequence analysis. BMC Genomics. 2011;12:444. doi:10.1186/1471-2164-12-444.
27. Moriya Y, Itoh M, Okuda S, Yoshizawa A and Kanehisa M. KAAS: an automatic genome annotation and pathway reconstruction server. Nucleic Acids Research. 2007;35:W182-W5.
