# Supplementary material for: Novel transcriptome resources for three scleractinian coral species from the Indo-Pacific
Source: Gigascience. 2017 Aug 23;6(9):1–4. doi: 10.1093/gigascience/gix074 (PMC5603760; doi:10.1093/gigascience/gix074)
Supplement: Response-to-Reviewer-Comments_Original-Submission.pdf [file gix074_Response-to-Reviewer-Comments_Original-Submission.pdf]

Author's Response To Reviewer Comments

Dear Dr. Zauner,

We would like to thank you, Dr. Kitchen and Dr. Hanna for your time and effort in reviewing our manuscript. We greatly appreciate the constructive comments and suggestions. Our responses to individual reviewer comments can be found below. We have numbered individual comments sequentially and our responses can be found following the ‘>>>>>>>>>>’ symbols.

Reviewer 1

1. The manuscript is well written but there is one section that after reviewing the details of Data Notes submission appears to be absent. According to GigaScience's guide to authors Data Notes should include, "information on data collection, detailed data validation, and information on exactly how these data can be re-used." The authors touch on the first two in detail but provide little information on how these data can be (re)used. An additional section following the "Evaluation of Assemblies" on the potential novel applications of these new data is needed to complete this manuscript. I believe this data is important to get out to the community, but minor revision is required.

>>>>>>>>>Thank you for catching this oversight. We have now added in a section on the potential for re-use as follows (L118-123): “These coral host-specific assemblies are sufficient for use as transcriptome references for Tag-based RNAseq (TagSeq) [30], a cost-effective method which was recently shown to be more accurate at quantifying gene expression levels than traditional RNAseq [31]. The fasta files and associated annotation files have been formatted for direct use in the TagSeq read mapping (https://github.com/z0on/tag-based_RNAseq) and GO-MWU analysis pipelines (https://github.com/z0on/GO_MWU)”.

2. Supplemental Figures 1-3: The use of color is misleading in these figures based on the figure legend description. When I pull up the Nematostella vectensis glycolysis /gluconeogenesis pathway (http://www.genome.jp/kegg-bin/show_pathway?nve00010), not all genes (boxes) are colored (ie. present in the Nematostella genome). However, based on the figures I would guess that the new transcriptomes only have a small fraction of the Nematostella homologs (more white boxes than shaded boxes). To accurately present the new data compared to the genes present in Nematostella, a separate fill color should be used for (1) Nematostella-only, (2) new transcriptome-only, and (3) those shared by the two resources. For example, 2.7.1.147 was not found in the G. astreata assembly but is present in Nematostella. Then it will be clear that the transcriptomes have complete or nearly-complete pathways, but might have slight differences in the genes present.

>>>>>>>>>We have elected to replace this analysis with a more comprehensive assessment of transcriptome completeness suggested by Reviewer 2. We have replaced the KEGG map figures with a comparison to the Benchmarking Universal Single-Copy Ortholog (BUSCO v2) [29] set for metazoans. Our assemblies contain complete copies of 90% of these orthologs, on average. We have revised the text as follows (L93-96): “Transcriptome completeness was evaluated through comparison to the Benchmarking Universal Single-Copy Ortholog (BUSCO v2) [29] set

for metazoans using the gVolante server (https://gvolante.riken.jp/analysis.html).” and (L114-116): “Of the 978 core BUSCO gene set for metazoans [29], 89.98-91.92% were found to be complete, while an additional 3.07-3.68% were partially assembled indicating that assemblies are fairly comprehensive (Table 1).”

3. Line 19: Remove "the first", which is written twice.

>>>>>>>>>Corrected.

4. Line 73: Italicize Acropora tenuis.

>>>>>>>>>Italicized.

5. Line 92: Provide the citation for the BBMap package.

>>>>>>>>>Citation added.

Reviewer 2

6. Overall the article is well written and easy to understand. I think they should add few sentences in the introduction, on why they chose these particular corals and why they heat-stressed them, it is a bit unclear to the reader (who might be outside the coral world) what was the motivation for this experiment.

>>>>>>>>>We pooled control and heat-treated RNA samples from the same individual to maximize transcript representation, given that coral gene expression is modified under thermal stress. We refer readers to the manuscript describing the motivation for the thermal stress experiment and have now added a clarification on sample pooling as follows (L44-48): “To generate more comprehensive reference transcriptomes, 4-5 replicate cores of a single colony were subject to a two-week temperature stress experiment as described in [18] and paired samples from control (27°C) and heat (31°C) treatments were snap frozen in liquid nitrogen on day 2, day 4 and day 17 (Table 1, note for G. acrhelia, heat-treated fragments only included for day 4 and day 17).”

7. Line 61: using a custom perl script … : I suggest to the authors to create a permanent branch/fork of the version that was used of the github repository. Since this is a git-based system, any commits to repository in the future might bring different results to those who try to re-run the analysis.

>>>>>>>>>Thank you for this suggestion, we have replaced the master link with a link to a forked version.

8. Line 73: "Acropora tenuis", should be in italics

>>>>>>>>>Italicized.

9. Figure S1,S2,S3 : I am not sure if these KEGG maps are needed. If the authors really want to show the completeness of their transcriptome, I recommend they utilize this tool https://gvolante.riken.jp/analysis.html , which would give an accurate measure of how many families they covered. Especially that there is no explanation why these three pathways were chosen, vs. for example other core metabolic pathways?

>>>>>>>>>Thank you for suggesting this alternative analysis. We have replaced the KEGG map figures with a comparison to the Benchmarking Universal Single-Copy Ortholog (BUSCO v2) [29] set for metazoans. Our assemblies contain complete copies of 90% of these orthologs, on average. We have revised the text as follows (L93-96): “Transcriptome completeness was evaluated through comparison to the Benchmarking Universal Single-Copy Ortholog (BUSCO v2) [29] set for metazoans using the gVolante server (https://gvolante.riken.jp/analysis.html).” and (L114-116): “Of the 978 core BUSCO gene set for metazoans [29], 89.98-91.92% were found to be complete, while an additional 3.07-3.68% were partially assembled indicating that assemblies are fairly comprehensive (Table 1).”

10. I assume the authors will include the assembled transcriptome with this gigascience data note, if not I recommend they upload them to other data sharing websites such dryad or figshare, which should guarantee more permanence and access to the assemblies.

>>>>>>>>> We have now also archived transcriptomes on the Australian Institute of Marine Science Data Centre which is a permanent, open access repository and have revised the section on Data Accessibility as follows (L126-131): “Raw reads are archived at NCBI’s SRA under project numbers PRJNA350363: Goniopora columna, PRJNA352640: Galaxea archelia, PRJNA352641: Galaxea astreata. The assembled transcriptomes and associated annotation files can be obtained from http://dornsife.usc.edu/labs/carlslab/data/ or from the Australian Institute of Marine Science Data Centre at http://data.aims.gov.au/metadataviewer/faces/view.xhtml?uuid=3c2d31c9-b921-491c-ae27-0d169fa98c84.”
