# Supplementary material for: Novel transcriptome resources for three scleractinian coral species from the Indo-Pacific
Source: Gigascience. 2017 Aug 23;6(9):1–4. doi: 10.1093/gigascience/gix074 (PMC5603760; doi:10.1093/gigascience/gix074)
Supplement: Response-to-Reviewer-Comments_Revision-1.pdf [file gix074_Response-to-Reviewer-Comments_Revision-1.pdf]

Author's Response To Reviewer Comments

Dear Dr. Zauner,

In recently re-reviewing my transcriptome assemblies during a separate analysis, I realized that isogroups (groups of transcript isoforms putatively originating from a single gene) were incorrectly clustered.

Trinity software designates assembled transcript clusters with a 3 part name, e.g 'c85276_g2_i1'. In prior software releases, the cluster designation (formerly labelled Tr) was synonymous with isogroup - e.g. c85276_g2_i1 corresponded with a transcript isoform assigned to isogroup85276.

I failed to realize that in the most current software release, unique isogroups are actually designated by the combination of the cluster ‘c’ and gene ‘g’ identifiers, e.g. in the above example, the cluster 'c85276_g2_i1' should be assigned to isogroup85276_g2

Consequently, my original annotation files had too few isogroups. I have redone the annotation files for my transcriptome assemblies to correct this error and have revised my manuscript to reflect these new annotation statistics.

Importantly, the assemblies themselves have not changed and the completeness metrics are unaltered, as these were based on the full transcriptome fasta. The only change is in how clusters have been assigned to isogroup designations and the subsequent gene name, gene ontology, KOG and KEGG annotations for the isogroups.

I have attached both the final manuscript draft and a track-changes version so you can see exactly what numeric values have been changed. The scripts available through the github link have also been updated to reflect this change and the transcriptome download files on my personal website have also been updated with the correct annotation files (and a note has been added to the README to indicate this update in addition to a general news posting on my site home page).

The transcriptome annotation files in the GigaDB dataset 100328 will also need to be updated to reflect these changes.

Again, my sincerest apologies for failing to catch this error prior to manuscript submission or in earlier review stages. Please let me know if you have any questions regarding these updates.

Sincerely,

Carly Kenkel
