# Supplementary material for: Novel transcriptome resources for three scleractinian coral species from the Indo-Pacific
Source: Gigascience. 2017 Aug 23;6(9):1–4. doi: 10.1093/gigascience/gix074 (PMC5603760; doi:10.1093/gigascience/gix074)
Supplement: Reviewer-1-Report-(Original-Submission).pdf [file gix074_Reviewer-1-Report-(Original-Submission).pdf]

Reviewer Report

Title: Novel transcriptome resources for three scleractinian coral species from the Indo-Pacific

Version: Original Submission **Date:** 4/13/2017

Reviewer name: Sheila Kitchen

Reviewer Comments to Author:

Summary:Kenkel and Bay describe three new coral transcriptomic resources that contribute both ecological and evolutionary significance to the field of coral biology. The inclusion of pooled RNA from coral fragments exposed to ambient and elevated temperatures also provides a more comprehensive collection of transcripts for future global expression profiling studies on thermal stress. The authors provided a comparison of the holobiont and host-specific assemblies, which is a necessary step for corals and allows for further investigation into host vs. symbiont (Symbiodinium and/or microbial) transcription patterns. Based on their evaluation of the transcriptomes, these new resources are comparable to other published scleractinian transcriptomes in contiguity (protein coverage >75%) and completeness (% of KOGs). Overall, the availability of these data will enhance studies on these interesting coral species.The manuscript is well written but there is one section that after reviewing the details of Data Notes submission appears to be absent. According to GigaScience's guide to authors Data Notes should include, "information on data collection, detailed data validation, and information on exactly how these data can be re-used." The authors touch on the first two in detail but provide little information on how these data can be (re)used. An additional section following the "Evaluation of Assemblies" on the potential novel applications of these new data is needed to complete this manuscript. I believe this data is important to get out to the community, but minor revision is required.Minor Comments:Supplemental Figures 1-3: The use of color is misleading in these figures based on the figure legend description. When I pull up the Nematostella vectensis glycolysis /gluconeogenesis pathway (http://www.genome.jp/kegg-bin/show_pathway?nve00010), not all genes (boxes) are colored (ie. present in the Nematostella genome). However, based on the figures I would guess that the new transcriptomes only have a small fraction of the Nematostella homologs (more white boxes than shaded boxes). To accurately present the new data compared to the genes present in Nematostella, a separate fill color should be used for (1) Nematostella-only, (2) new transcriptome-only, and (3) those shared by the two resources. For example, 2.7.1.147 was not found in the G. astreata assembly but is present in Nematostella. Then it will be clear that the transcriptomes have complete or nearly-complete pathways, but might have slight differences in the genes present.Line 19: Remove "the first", which is written twice.Line 73: Italicize Acropora tenuis.Line 92: Provide the citation for the BBMap package.

Level of Interest

Please indicate how interesting you found the manuscript: An article whose findings are important to those with closely related research interests

Quality of Written English

Please indicate the quality of language in the manuscript: Acceptable

Declaration of Competing Interests

Please complete a declaration of competing interests, considering the following questions:

- Have you in the past five years received reimbursements, fees, funding, or salary from an organisation that may in any way gain or lose financially from the publication of this manuscript, either now or in the future?
- Do you hold any stocks or shares in an organisation that may in any way gain or lose financially from the publication of this manuscript, either now or in the future?
- Do you hold or are you currently applying for any patents relating to the content of the manuscript?
- Have you received reimbursements, fees, funding, or salary from an organization that holds or has applied for patents relating to the content of the manuscript?
- Do you have any other financial competing interests?
- Do you have any non-financial competing interests in relation to this paper?

If you can answer no to all of the above, write 'I declare that I have no competing interests' below. If your reply is yes to any, please give details below.

I declare that I have no competing interests.

I agree to the open peer review policy of the journal. I understand that my name will be included on my report to the authors and, if the manuscript is accepted for publication, my named report including any attachments I upload will be posted on the website along with the authors' responses. I agree for my report to be made available under an Open Access Creative Commons CC-BY license (http://creativecommons.org/licenses/by/4.0/). I understand that any comments which I do not wish to be included in my named report can be included as confidential comments to the editors, which will not be published.

I agree to the open peer review policy of the journal
