# Supplementary material for: Novel transcriptome resources for three scleractinian coral species from the Indo-Pacific
Source: Gigascience. 2017 Aug 23;6(9):1–4. doi: 10.1093/gigascience/gix074 (PMC5603760; doi:10.1093/gigascience/gix074)
Supplement: Reviewer-2-Report-(Original-Submission).pdf [file gix074_Reviewer-2-Report-(Original-Submission).pdf]

Reviewer Report

Title: Novel transcriptome resources for three scleractinian coral species from the Indo-Pacific

Version: Original Submission **Date:** 5/21/2017

Reviewer name: Bishoy Hanna

Reviewer Comments to Author:

The authors report on the transcriptome sequencing of three closely related corals Galaxea astreata and G. acrhelia and Goniopora columna. The data reported is important due to the dearth transcriptomic information from corals in general (compared to other taxa), and in particular Goniopora columna. This should enable future studies of these corals utilizing the transcriptomic data reported.Overall the article is well written and easy to understand. I think they should add few sentences in the introduction, on why they chose these particular corals and why they heat-stressed them, it is a bit unclear to the reader (who might be outside the coral world) what was the motivation for this experiment.The authors have taken all proper measures for data collection and analysis.Line 61: using a custom perl script … : I suggest to the authors to create a permanent branch/fork of the version that was used of the github repository. Since this is a git-based system, any commits to repository in the future might bring different results to those who try to re-run the analysis.Line 73: "Acropora tenuis", should be in italicsFigure S1,S2,S3 : I am not sure if these KEGG maps are needed. If the authors really want to show the completeness of their transcriptome, I recommend they utilize this toolhttps://gvolante.riken.jp/analysis.html , which would give an accurate measure of how many families they covered. Especially that there is no explanation why these three pathways were chosen, vs. for example other core metabolic pathways?I assume the authors will include the assembled transcriptome with this gigascience data note, if not I recommend they upload them to other data sharing websites such dryad or figshare, which should guarantee more permanence and access to the assemblies.

Level of Interest

Please indicate how interesting you found the manuscript: An article of importance in its field

Quality of Written English

Please indicate the quality of language in the manuscript: Acceptable

Declaration of Competing Interests

Please complete a declaration of competing interests, considering the following questions:

- Have you in the past five years received reimbursements, fees, funding, or salary from an organisation that may in any way gain or lose financially from the publication of this manuscript, either now or in the future?
- Do you hold any stocks or shares in an organisation that may in any way gain or lose financially from the publication of this manuscript, either now or in the future?
- Do you hold or are you currently applying for any patents relating to the content of the manuscript?
- Have you received reimbursements, fees, funding, or salary from an organization that holds or has applied for patents relating to the content of the manuscript?
- Do you have any other financial competing interests?
- Do you have any non-financial competing interests in relation to this paper?

If you can answer no to all of the above, write 'I declare that I have no competing interests' below. If your reply is yes to any, please give details below.

I declare that I have no competing interests.

I agree to the open peer review policy of the journal. I understand that my name will be included on my report to the authors and, if the manuscript is accepted for publication, my named report including any attachments I upload will be posted on the website along with the authors' responses. I agree for my report to be made available under an Open Access Creative Commons CC-BY license (http://creativecommons.org/licenses/by/4.0/). I understand that any comments which I do not wish to be included in my named report can be included as confidential comments to the editors, which will not be published.

I agree to the open peer review policy of the journal
